# Time of Day and a Ketogenic Diet Influence Susceptibility to SUDEP in *Scn1a*^*R1407X*/+^ Mice

**DOI:** 10.3389/fneur.2019.00278

**Published:** 2019-03-29

**Authors:** Frida A. Teran, YuJaung Kim, Megan S. Crotts, Eduardo Bravo, Katlynn J. Emaus, George B. Richerson

**Affiliations:** ^1^Department of Neurology, University of Iowa, Iowa City, IA, United States; ^2^Medical Scientist Training Program, University of Iowa, Iowa City, IA, United States; ^3^Iowa Neuroscience Institute, University of Iowa, Iowa City, IA, United States; ^4^Department of Biomedical Engineering, University of Iowa, Iowa City, IA, United States; ^5^Department of Molecular Physiology & Biophysics, University of Iowa, Iowa City, IA, United States; ^6^Neurology, Veterans Affairs Medical Center, Iowa City, IA, United States

**Keywords:** epilepsy, seizure, ketogenic diet, SUDEP, breathing, sleep, circadian

## Abstract

Sudden unexpected death in epilepsy (SUDEP) is a major cause of mortality in patients with drug-resistant epilepsy. Most SUDEP cases occur in bed at night and are preceded by a generalized tonic-clonic seizure (GTCS). Dravet syndrome (DS) is a severe childhood-onset epilepsy commonly caused by mutations in the *SCN1A* gene. Affected individuals suffer from refractory seizures and an increased risk of SUDEP. Here, we demonstrate that mice with the *Scn1a*^*R1407X*/+^ loss-of-function mutation (DS) experience more spontaneous seizures and SUDEP during the early night. We also evaluate effects of long-term ketogenic diet (KD) treatment on mortality and seizure frequency. DS mice showed high premature mortality (44% survival by P60) that was associated with increased spontaneous GTCSs 1–2 days prior to SUDEP. KD treated mice had a significant reduction in mortality (86% survival by P60) compared to mice fed a control diet. Interestingly, increased survival was not associated with a decrease in seizure frequency. Further studies are needed to determine how KD confers protection from SUDEP. Moreover, our findings implicate time of day as a factor influencing the occurrence of seizures and SUDEP. DS mice, though nocturnal, are more likely to have SUDEP at night, suggesting that the increased incidence of SUDEP at night in may not be solely due to sleep.

## Introduction

Sudden unexpected death in epilepsy (SUDEP) is estimated to occur in approximately 27% of patients with epilepsy (1). This number can increase to 50% in patients with poorly controlled and severe epilepsy (2). Although the mechanisms underlying SUDEP are not fully understood, an increasing body of evidence suggests SUDEP is due to seizure-induced cardiorespiratory arrest (3, 4). However, little is known about the circumstances leading up to SUDEP. A strong association with sleep has been documented in a number of studies (3, 5). Although a significant majority of patients are found in bed in the prone position at the time of death (6–8), the occurrence of SUDEP during sleep varies widely among published case studies (9). This suggests that circadian or other factors may be involved rather than time of day having an effect strictly due to sleep stage.

Many types of epilepsy have a substantial genetic component. Channelopathies involving the neuronal voltage-gated sodium channel *SCN1A* result in a wide spectrum of epilepsy phenotypes ranging from febrile seizures to Dravet Syndrome (DS) (10, 11). DS is a devastating epileptic encephalopathy of childhood-onset that typically manifests as febrile seizures in the first year of life and progresses to refractory epilepsy (12). In patients with DS, the risk of SUDEP is estimated to be 15 times higher than in other pediatric epilepsies (13). Premature death occurs in 21% of DS patients, with SUDEP accounting for nearly half of these deaths (14). Children with DS develop several comorbidities, such as ataxia, cognitive impairments, and sleep disturbances (11). Murine models of DS have proven to be an effective research tool for understanding the pathophysiology of SUDEP as they recapitulate many aspects of the clinical condition: they have heat-induced seizures, spontaneous seizures and a high incidence of premature mortality due to SUDEP (15). Notably, these mice also display impaired sleep architecture homeostasis (16).

A recent study found that time of day can have an independent influence on physiological changes associated with a seizure, particularly breathing (17). This is important as seizure-induced changes in respiratory physiology contribute to SUDEP in patients (3, 18–24) and in DS mice (15). In the present study, we aimed to determine in DS mice whether: (1) spontaneous seizures and SUDEP are more likely to occur in the light or dark phase; (2) seizure frequency changes in the days prior to SUDEP; and (3) treatment with a high-fat, low-carbohydrate ketogenic diet (KD), which has been proven to be protective in other seizure models (25, 26), results in fewer spontaneous seizures and SUDEP.

## Materials and Methods

### Mouse Husbandry and Genotyping

A pair of *Scn1a*^*R1407X*/+^ heterozygous male mice on a C3HFeB/HeJ background were provided by Miriam Meisler (University of Michigan, Ann Arbor, Michigan, USA), and were bred with C3HFeB/HeJ female mice (Jackson Laboratory) to establish a breeding colony. *Scn1a*^*R1407X*/+^ mice are referred to as “DS mice” for the entirety of this manuscript. Breeding and genotyping of these mice have been previously described (27). Briefly, DS mice were genotyped by PCR amplification with the primers DS-F (5′ CAATGATTCCTAGGGGGATGTC 3′) and DS-R (5′ GTTCTGTGCACTTATCTGGATTCAC 3′). Genomic DNA was PCR amplified, digested with HpaII, and separated on 2% agarose gels containing 0.15 μg/ml ethidium bromide. Digestion of the PCR product with HpaII generated 2 fragments (295 bp and 223 bp) from the WT allele and an uncut fragment (518 bp) from the mutant allele. DS mice were housed in a 12:12 h light-dark regimen (lights on 7:00 a.m. to 7:00 p.m.) in standard cages with food and water available *ad libitum*. Body weight was monitored weekly from the time of weaning (P21) until P60.

### Diet Groups

DS mice were randomly weaned onto either a control diet consisting of standard chow (7013, Teklad Diets, Madison, WI, U.S.A) or a KD (Bio-Serv F3666, Frenchtown, NJ, U.S.A.) (see Table 1).

**Table 1:** Composition of Diets.

	**Control (TD 7013)**		**KD (F3666)**	
	**% by weight**	**% kcal from**	**% by weight**	**% kcal from**
Protein	20	23	11.4	4.8
Carbohydrate	64.8	59	10.8	1.8
Fat	8.2	18	77.8	93.4
Kcal/g		3.1		7.24
F:P+C		0.1:1		6.3:1
Animals (*N*)		124		66

### Monitoring of Spontaneous Seizures and Deaths in Mice

DS mice were housed in their home cages under continuous video surveillance to monitor for spontaneous seizures and deaths from P16-P60 as previously described (15). Briefly, video recordings were made at 30 frames per second using web cameras with night vision (FL8910W; Foscam Digital Technologies). Up to 32 cameras were connected to a single computer, and video recordings were saved in 8-h segments and stored on an external hard drive using commercial video webcam software (Blue Iris 4; Foscam Digital Technologies). When a mouse was found dead in a cage, the video was reviewed to determine time of death and whether death was preceded by a behavioral seizure. In the light-dark cycle, light phase was defined as the period between 07:01 and 19:00 h, whereas the dark phase refers to the period between 00:01–07:00 and 19:01–24:00 h.

### Seizure Semiology

Animal seizure activity and deaths were assessed by video review by an observer blind to the diet groups. Seizures detected during video review were scored using a modified Racine scale (28). To ensure consistency of seizure classification, only spontaneous seizures scoring 4 (rearing with forelimb clonus and loss of postural control, bilateral myoclonus, and/or wild running and jumping) or 5 (tonic hindlimb extension) were documented.

### β-HB Measurement

To determine whether the ketogenic diet increased circulating levels of ketone bodies in mice, blood samples were collected from P35-40 DS mice randomly selected from each diet group to test for the ketone body beta-hydroxybutyrate (β-HB). Animals were anesthetized with a Ketamine/Xylazine cocktail (87.5 mg/kg Ketamine/12.5 mg/kg Xylazine, IP). Blood samples were collected via cardiac puncture into an EDTA pre-coated syringe to prevent coagulation and centrifuged at 3,500 rpm, 4°C, for 5 min to obtain plasma. β-HB levels were determined in duplicate using a commercially available enzyme colorimetric β-HB Assay kit (BioVision, Mountain View, CA). OD450 readings were determined using plate spectrophotometry (BioTek Synergy 4, Winooski, VT).

### Statistical Analysis

Statistical analysis was performed with Prism 8 (GraphPad Software, Inc., La Jolla, CA, U.S.A.). Comparisons across groups were done with unpaired two-tailed *t*-tests and differences across time points were determined using repeated measures one-way analysis of variance (ANOVA) with appropriate *post hoc* test if indicated as noted. A two-way ANOVA was used to evaluate seizure frequencies between and within light/dark phases and diet groups. Survival curves were constructed using the Kaplan-Meier method and comparisons made with the Log-rank test. All data points are presented as averages ± standard error of the mean (SEM) unless noted. Significance was set at *P* < 0.05.

## Results

### DS Mice Had a Higher Incidence of SUDEP From Late Evening to the Middle of the Night

**Figure 1:**
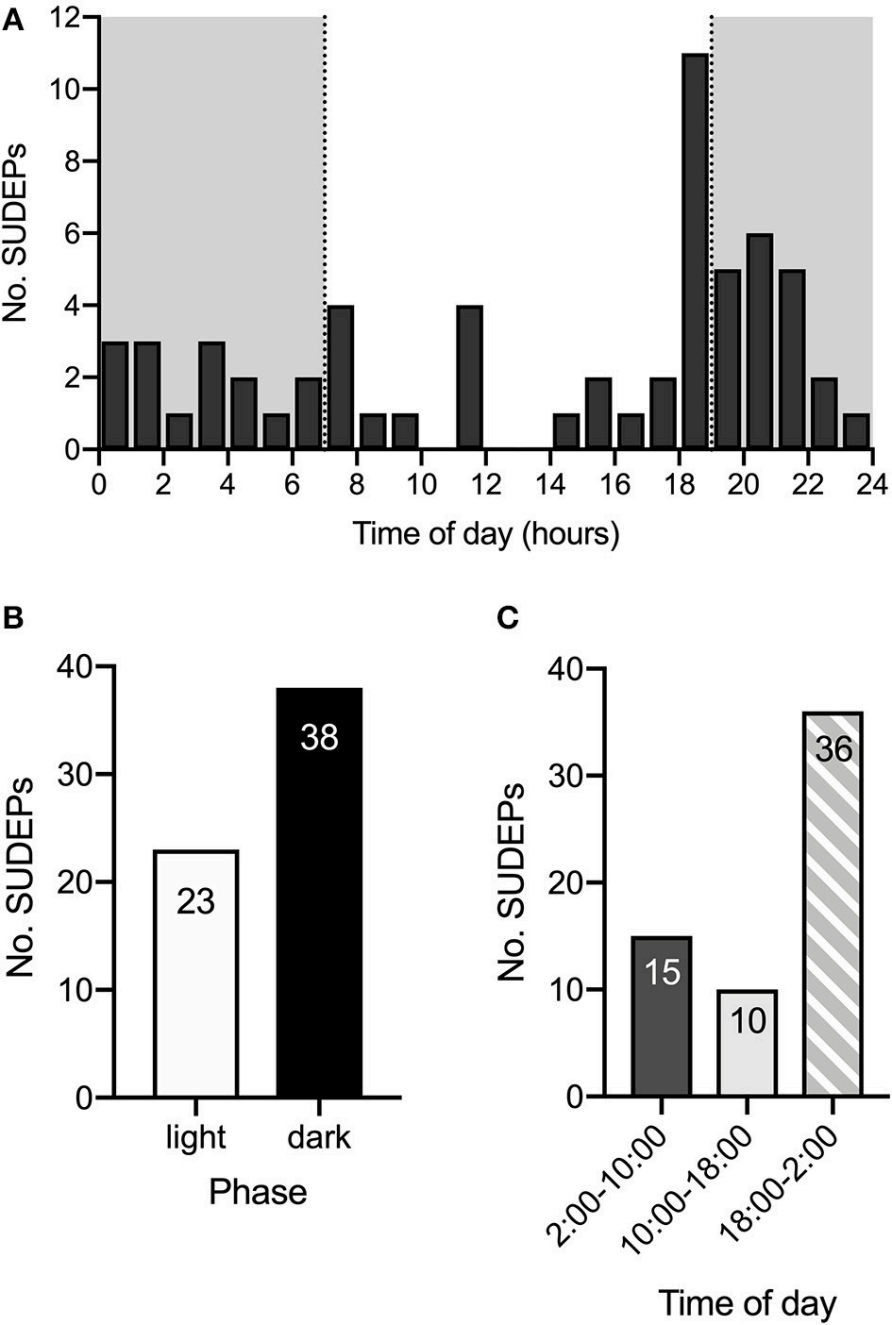
SUDEP is more common during late evening and early night. **(A)** Histogram of time of SUDEP (*n* = 61) across the 24-h day for *Scn1a*^*R1407X*/+^ mice. The dark phase is denoted by the gray background. **(B)** SUDEP was more common during the dark phase than the light phase in *Scn1a*^*R1407X*/+^ mice. **(C)** When the 24-h day was divided into 8-h segments, there were more SUDEPs that occurred between 18:00 and 02:00 than the other two segments. Data were obtained from DS mice fed a control diet.

To determine the time of day at which spontaneous deaths are most likely to occur, long-term video surveillance was maintained starting between P16 & 21 for all mice and continued until P60 or until death to monitor for spontaneous seizures and sudden deaths. SUDEPs in 61 DS mice on a control diet were captured on video. Review of video recordings revealed that all deaths occurred after a GTCS with hindlimb extension (Racine scale 5), similar to previous observations (15, 29). The time of death was determined and a histogram of number of deaths vs. time of day (Figure 1A) revealed that SUDEP predominantly occurred in late evening (18:00–19:00) or in the first part of the dark phase between 19:00 and 05:00. The total number of deaths that occurred in the dark phase was 1.65-fold greater than the number in the light phase (38 vs. 23) (Figure 1B). However, the number of deaths peaked just before the lights went out and remained high predominantly during the early part of the night. Taking this into account, when the day was divided into 8-h segments, the number of SUDEPs between 18:00 and 02:00 (36) was 3.6-fold greater than those between 10:00 and 18:00 (10) and 2.4-fold greater than between 02:00 and 10:00 (15) (Figure 1C).

### DS Mice Had a Higher Incidence of Spontaneous Seizures in Late Evening and Early Night

**Figure 2:**
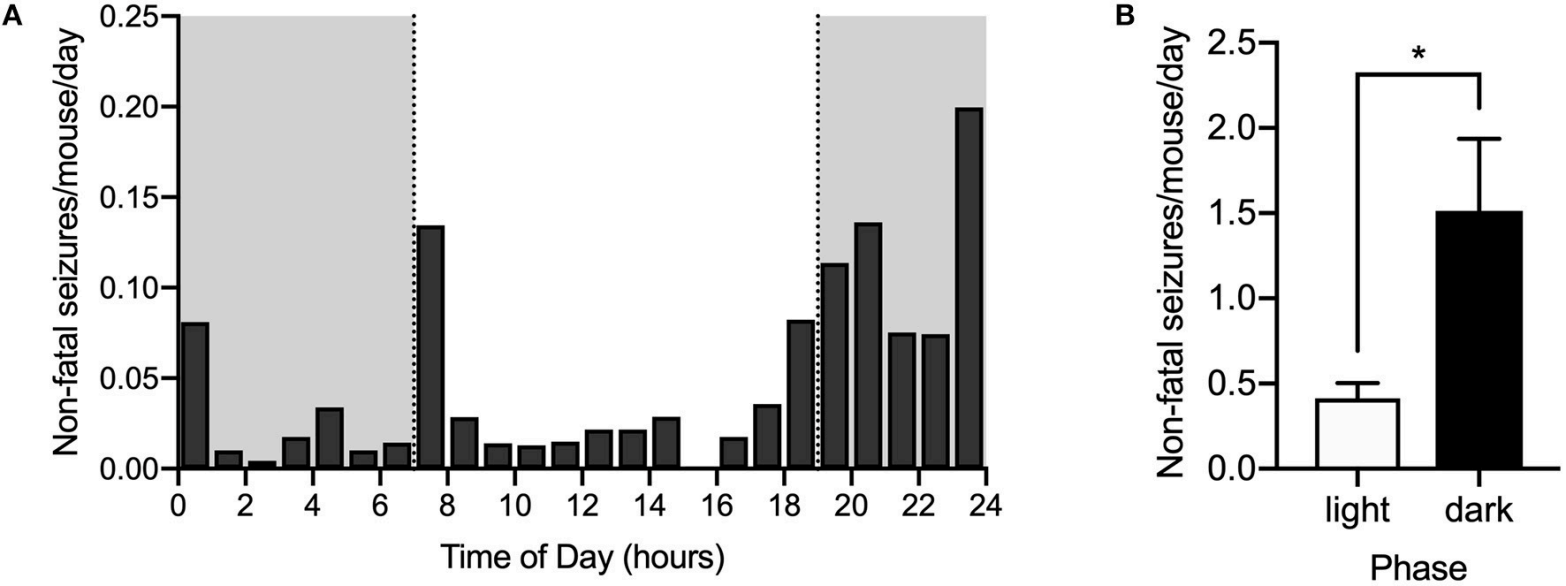
Seizures are also more common during late evening and early night. The number of non-fatal seizures per day for each mouse was calculated as described in the text based on monitoring video recordings. **(A)** Histogram of spontaneous convulsive non-fatal seizures (Racine 4 & 5) per day per mouse distributed across the 24-h day. Dark phase is denoted by the gray rectangles (*n* = 21 mice from 13 cages). **(B)** Seizures were more frequent in the dark phase than in the light phase (^*^p = 0.005, Wilcoxon matched-pairs signed rank test). Data were obtained from DS mice fed a control diet.

To determine the time of day during which spontaneous seizures are most likely to occur, video recordings from 21 DS mice randomly selected from the control diet group in 13 cages (1.62 ± 0.87 mice per cage, mean ± SD) were reviewed starting at an age of P20 and continuing for 6.38 ± 2.96 days (mean ± SD). A total of 121 spontaneous non-fatal seizures were captured on video. Since some cages had up to three mice and individuals could not always be identified consistently throughout the time of recording, the number of seizures recorded from each cage was divided by the number of mice per cage and then by the number of days recorded, to obtain an estimate of the average number of seizures per day for each mouse. Non-fatal seizures also occurred in late evening (18:00–19:00) and during the early part of the night (19:00–01:00) (Figure 2A), similar to the distribution of SUDEP. There were more than three times as many seizures per mouse in the dark phase (1.51 ± 0.42) than in the light phase (0.41 ± 0.09) (*p* = 0.005, Wilcoxon matched-pairs signed rank test) (Figure 2B). Similar to what was found for SUDEP, the difference was even greater when the number of seizures was determined between 18:00 and 01:00 compared to between 01:00 and 18:00.

### Seizure Frequency Increased the Day Prior to Death

**Figure 3:**
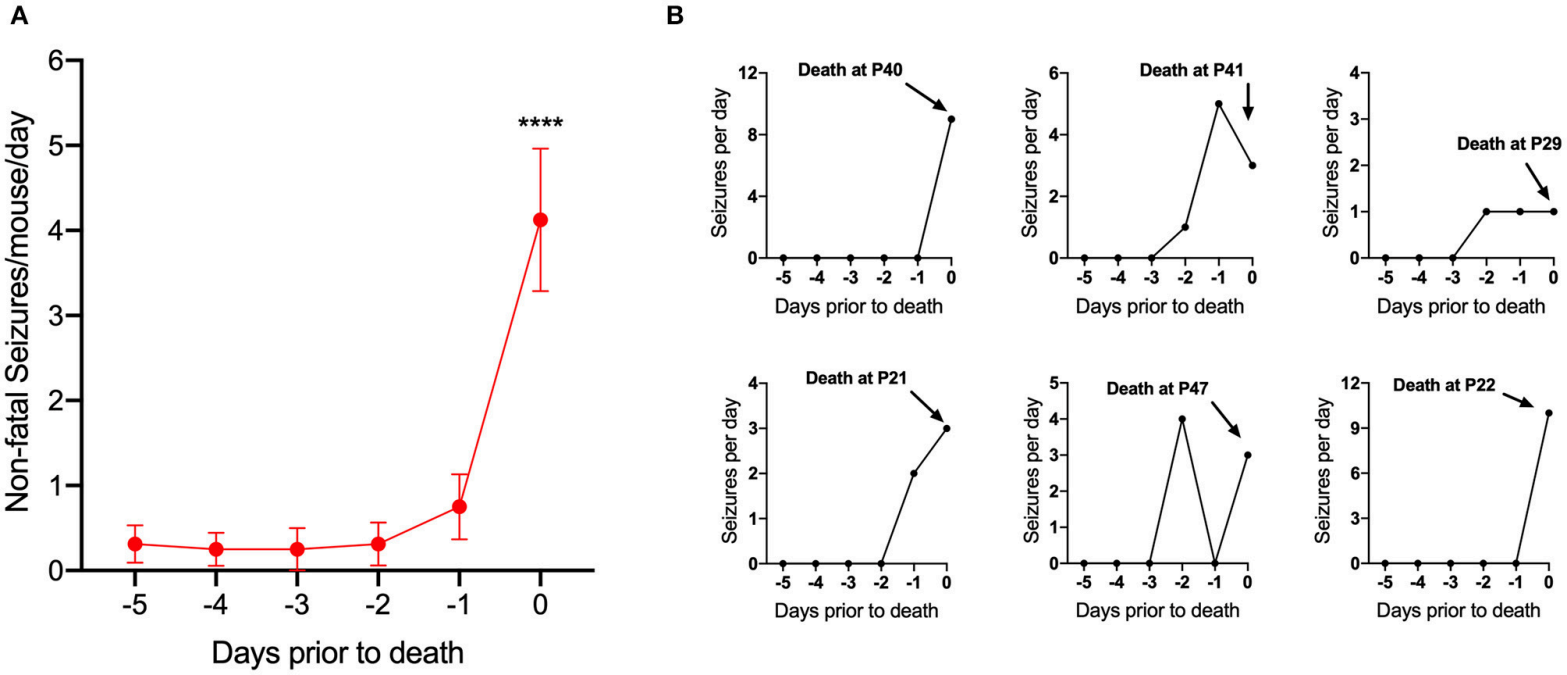
Seizure frequency increased 1–2 days prior to death. **(A)** Number of non-fatal convulsive seizures per mouse per day plotted as a function of days prior to death (*d* = −1 was 24 h prior to death). Seizures significantly increased during the last 1–2 days prior to death [*F*_(5, 90)_ = 13.51, *p* < 0.0001] (*n* = 16 mice). ^****^p < 0.0001. **(B)** Representative examples of spontaneous seizure progression for 5 days prior to SUDEP in 6 *Scn1a*^*R1407X*/+^ mice.

To determine the relationship between seizure frequency and death, 16 DS mice that died of SUDEP (at any age) while on a control diet were randomly selected and video recordings of the last 5 days prior to death were reviewed. For each mouse, days before death were defined as 24-h consecutive periods prior to the time of death. Fatal seizures in DS mice followed a stereotypical progression: seizures began with forelimb clonus (Racine 3) followed by rearing and falling to the side (Racine 4), eventually leading to a GTCS ending with tonic hindlimb extension (Racine 5). All deaths occurred immediately following Racine 5 seizures, which we previously reported to be due to terminal apnea followed by bradycardia (15). Most mice had few or no spontaneous seizures until the last 1–2 days prior to death [*F*_(5, 90)_ = 13.51, *p* < 0.0001] (Figure 3). A run-up of seizures prior to death has been reported previously but was only examined during the last day before death (29).

### KD Treatment Reduced Mortality in DS Mice

**Figure 4:**
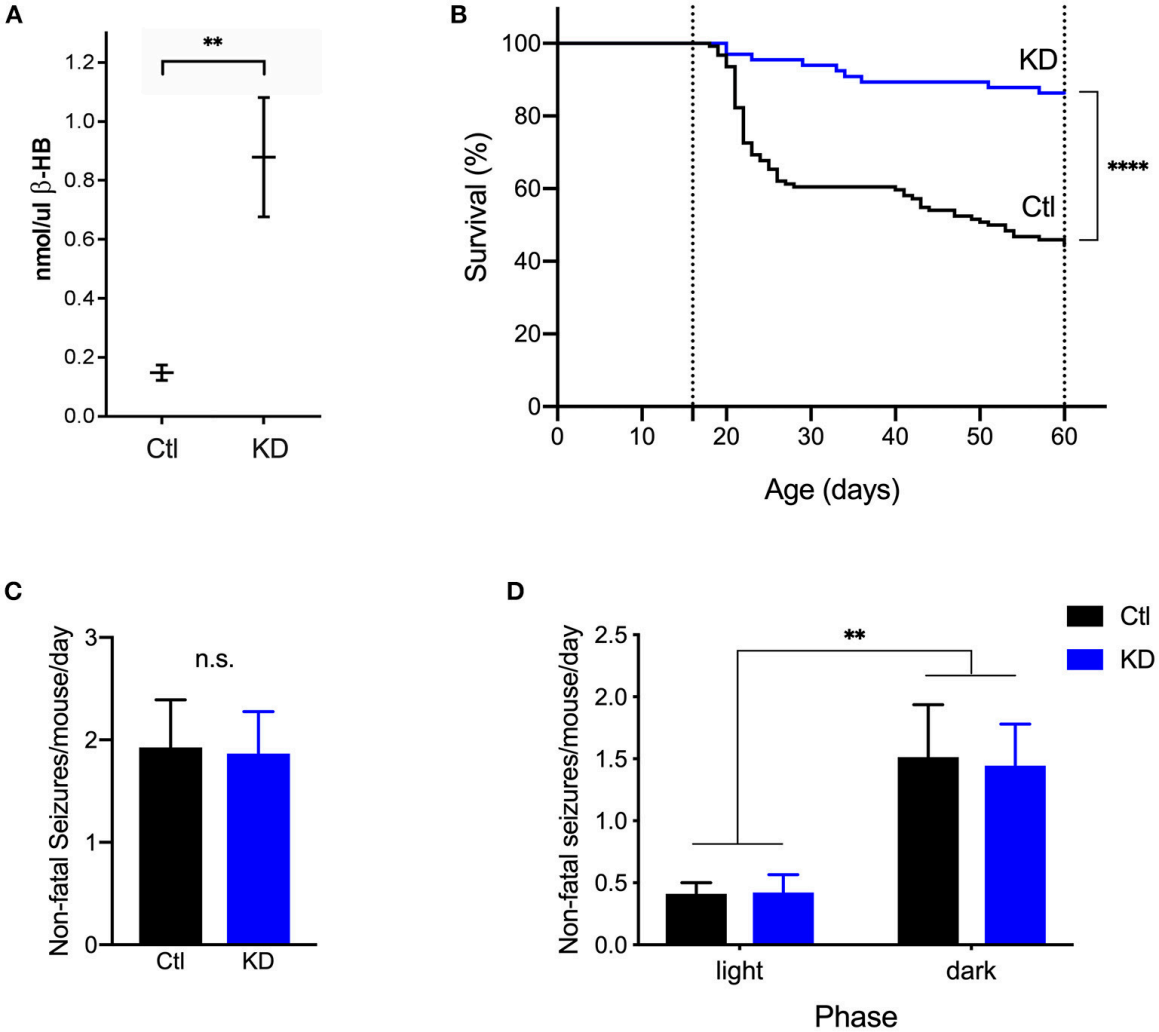
The ketogenic diet reduces mortality in *Scn1a*^*R1407X*/+^ mice. **(A)** As expected, beta-hydroxybutyrate (β-HB) levels were significantly increased in DS mice treated with a KD (*n* = 7) compared to control (Ctl, *n* = 6) (^**^p = 0.0012, Mann-Whitney U test). **(B)** Video surveillance revealed that 55% (*n* = 69 of 124) of DS mice fed a control diet (Ctl) spontaneously died after a generalized seizure with tonic hindlimb extension between P16 and P60. Treatment with a KD significantly increased survival of DS mice (KD, *n* = 57 of 66). ^****^p < 0.0001. **(C)** There was no difference in the frequency of seizures between diet groups. **(D)** Both diet groups experienced more seizures in the dark phase than in the light phase [*F*_(1, 28)_ = 10.95, *p* = 0.0026]. (*n* = 9 mice from 4 cages for KD, *n* = 21 mice from 12 cages for Ctl).

To test the effect of the KD on survival and seizures, a separate group of DS mice (*n* = 66) was fed a KD in parallel with control-fed mice. Blood was sampled from a randomly selected subset of mice (P35-40) from each diet group (KD, *n* = 7; control, *n* = 6) to measure levels of β-HB. As expected, KD-treated mice had significantly higher circulating levels of β-HB compared to control (*p* = 0.001, Mann-Whitney U test) (Figure 4A). Long-term video surveillance was maintained as described above. During that time, 56% of DS mice on a control diet (*n* = 124) died of SUDEP (Figure 4B). As we have previously reported, there was no difference in mortality between male and female mice (15). KD treatment (*n* = 66) significantly increased survival by approximately 42% (from 44 to 86%, *p* < 0.0001, Log-rank test) when compared to mice fed a control diet (Figure 4B). Since only 7 deaths were recorded in the KD group, we were not able to determine whether deaths in KD-treated mice occurred more often at night (4 deaths) than during the day (3 deaths).

We next assessed the effect of the KD on seizure frequency. To this end, video recordings from 9 randomly selected mice in 4 cages (2.25 ± 0.96 mice per cage, mean ± SD) from the KD group were reviewed starting at an age of P20 and continuing for 5 days per mice. A total of 40 spontaneous, non-fatal seizures in KD-treated mice were captured on video. The average number of seizures per day for each mouse was calculated as described above. KD-treated mice had an average of 1.867 ± 0.409 seizures per day, but when compared to control mice (1.925 ± 0.465), the difference was not statistically significant (*p* = 0.2766, Mann-Whitney U test) (Figure 4C). We then determined the time of day during which spontaneous seizures were most likely to occur. KD-treated mice experienced more seizures in the dark phase (1.44 ± 0.34) than in the light phase (0.42 ± 0.14) (*p* = 0.0430, Wilcoxon matched-pairs signed rank test) Taken together, both diet groups had more seizures in the dark phase than in the light phase [*F*_(1, 28)_ = 10.95, *p* = 0.0026], but no differences within each phase were found between the KD and control group (*p* = 0.939) (Figure 4D).

## Discussion

In the current study, we demonstrated that *Scn1a*^*R1407X*/+^ mice had a high incidence of premature mortality that was associated with an increased incidence of spontaneous GTCSs 1 day prior to death. We also made the novel observation that DS mice treated with a KD had a significant reduction in mortality compared to mice fed a control diet, but surprisingly this was not associated with a decreased incidence of spontaneous seizures. Spontaneous seizures and SUDEP in DS mice occurred more frequently at night, suggesting that time of day influences seizures and their outcome, but unlike epilepsy patients SUDEP was more common in mice during the time of day when they are more likely to be awake and active.

### Spontaneous Seizures and SUDEP Were Influenced by Time of Day in *Scn1a^*R1407X*/+^* Mice

A consistent factor in human SUDEP cases is that they occur more frequently at night (5, 9, 30), and it is widely assumed that death occurs during the sleep state. However, the specific mechanisms that cause SUDEP to occur at night are unknown, and it is possible that nighttime prevalence is due to circadian effects, the physical environment of being in bed, lack of supervision, or some other factor rather than being due to sleep. Here we made the novel observation that spontaneous seizures and SUDEP in *Scn1a*^*R1407X*/+^ mice occur more often at night, but since mice are nocturnal they are more likely to be awake at that time.

It still remains possible that sleep state is an independent risk factor for seizure occurrence and mortality. Although mice are nocturnal, their sleep is highly fragmented, and they have frequent short sleep bouts even at night. There is state-dependent variability in cardiac and respiratory function (31–33), which is important as SUDEP in *Scn1a*^*R1407X*/+^ mice is due to seizure-induced respiratory arrest (15). Sleep state can also influence the frequency and severity of seizures (34). Previous studies found that seizures induced via maximal electroshock (MES) during sleep were more likely to be fatal (5, 17). Although the vigilance state during which non-fatal and fatal seizures occurred was not determined in our study, seizures and SUDEP in our DS mice predominantly happened at night when mice are mostly awake. That SUDEP occurs mostly at night could also implicate a circadian influence, changes in motor activity, effects of light, or other entrained variables such as body temperature.

Interestingly, our data indicate that the peak incidence of seizures and SUDEP occurred in late evening before the lights went out (18:00–19:00) and ended before transitioning back to light phase between 01:00 and 05:00, suggesting it was not strictly the light/dark cycle that dictated risk of death. Instead, mice were more likely to die during the transition from the period of sleep (during the day for mice) to the period of increased motor activity (during early night). Nevertheless, major circadian abnormalities including a longer circadian period and severely impaired circadian photoresponsiveness have been identified in *Scn1a*^+/−^ mice (35). Whether intrinsic circadian deficits in DS mice play a role in seizure occurrence or SUDEP is yet to be explored. One way to address this could involve manipulating light-dark cues or maintaining animals in constant darkness to assess the relationship between seizures and SUDEP and their intrinsic free-running circadian clock.

### There Was a Large Increase in Seizure Frequency in the 24 h Prior to SUDEP

The circumstances surrounding SUDEP cases remain elusive, but it is believed that most deaths are preceded by a GTCS (18). A previous study reported a progression of increasingly severe spontaneous seizures preceding death in *Scn1a*^+/−^ mice, but seizures were monitored for only 24 h prior to death (29). In the present study, the last 5 days prior to death were reviewed. Most mice had few or no spontaneous seizures 3–6 days before SUDEP, but there was a surge in the frequency of seizures in the last 1–2 days prior to death. Interestingly, we did not observe a consistent pattern. For instance, one mouse had one GTCS that was fatal, whereas another had 10 non-fatal seizures prior to the final one that resulted in death. The reasons underlying this variability in the number of GTCS leading up to death are unknown.

A recent report of three SUDEP cases showed that no convulsive seizures or abnormal electroencephalographic (EEG) activity were observed prior to death (36). However, the absence of cortical EEG activity does not exclude hidden seizures that may have contributed to the fatal cascade. The cardiorespiratory collapse observed in these and other witnessed SUDEP cases implicates a brainstem mechanism, such as the spreading depolarization initiated by high potassium or tetanic neuronal stimulation in mice (37). Since our mice were not instrumented, EEG activity was not assessed. This limited our detection threshold to convulsive seizures. Whether non-seizure SUDEP ever occurs in DS mice is not known. Nevertheless, all sudden deaths we documented were preceded by behavioral seizures.

### Effect of a Ketogenic Diet on SUDEP and Seizures in *Scn1a^*R1407X*/+^* Mice

Previous studies have shown that a KD increases seizure threshold in multiple models of inducible seizures (38), including flurothyl-induced seizures in heterozygous *Scn1a* knockout (*Scn1a*^+/−^) mice (39). A KD has also been shown to reduce spontaneous seizures and extend the lifespan of *Kcna1*-null mice, a model of early onset epilepsy and SUDEP (25, 40). To our knowledge, the present study is the first to assess the effect of chronic treatment with a KD on spontaneous seizures and sudden death in *Scn1a*^*R1407X*/+^ mice. Herein, we report that a KD markedly increases survival in DS mice to P60 from 44 to 86%. Since previous work has shown anticonvulsant effects of a KD, we hypothesized that reduced mortality in KD-treated mice was associated with a decreased incidence of spontaneous seizures. To our surprise, no significant differences in seizure frequency or severity were observed between diets. This suggests that the protection conferred by the KD on mortality in our DS mice is not due to an antiseizure effect. One possibility is that the KD prevents the propagation of seizures from the forebrain to brainstem nuclei that are critical for cardiorespiratory control (21). This could be explained by recent findings showing that KD-treatment in rodents rescued cytological and molecular correlates of chronic epilepsy, such as cortical gliosis and cell loss in the CA1 and CA3 hippocampal areas seen in chronic temporal lobe epilepsy (41). However, the effects of the KD on brain cytology were not explored in this study.

### What Is the Link Between Seizures and SUDEP?

An important question is, what are the circumstances that lead to a seizure becoming fatal? In the present study, KD treatment prevented death without affecting the frequency of seizures, which challenges the notion that uncontrolled GTCS are the strongest risk factor for SUDEP. One possibility is that KD-treatment may stabilize seizure-induced respiratory changes by indirect mechanisms, thus preventing fatal apnea. How dietary manipulations such as the KD influence respiratory physiology has not been explored.

A related question is why are seizures and SUDEP both tied to time of day if seizures do not always lead to death? The answer to this question will require a better understanding of what factors link seizures and SUDEP to time of day.

### Limitations

A limitation to our study is the breadth of days and ages analyzed, as for most of our experiments we only reviewed video recordings from P20 to ~P26. We chose this age range because that is when many spontaneous deaths occur (Figure 4B), so that it might be expected that there would be a greater likelihood of cardiorespiratory dysregulation. In addition, studying a cohort of older mice would exclude all mice that had died at an earlier age—a group that may have more cardiorespiratory abnormalities. It is possible that seizure frequency is affected by the KD at a later age, which would be consistent with previous observations from *Kcna1*-null mice (25).

The mechanism by which a KD protects DS mice from SUDEP is unclear. Since seizure frequency was determined visually in uninstrumented animals, only seizures scoring 4 and above in a modified Racine scale were documented. Very few KD-treated DS mice died of SUDEP, therefore we have not recorded cardiorespiratory parameters during SUDEP in any of that group. Further studies with EEG monitoring and plethysmography will be necessary to determine whether terminal events, such as GTCS and apnea, are altered by the KD.

### Clinical Relevance

To this day, about twenty-seven FDA-approved AEDs are available, yet more than one third of epilepsy patients have inadequate control of seizures. As a result, the use of KDs has resurfaced in past decades to treat refractory epilepsy. Because administration and adherence to the strict KD are difficult for both patients and caregivers, elucidating the protective mechanism of the KD has increasingly become a pursuit of great interest for clinical and basic research.

## Conclusion

The unpredictable and often unwitnessed occurrence of SUDEP presents a challenge to investigators and clinicians in research studies on patients. DS mouse models provide an efficient research tool for understanding the pathophysiology of SUDEP and developing effective therapies as they recapitulate human DS. Here we use a murine DS model to show the relationship between time of day and SUDEP, and an unexpected lack of effect of a KD on seizures despite protection against SUDEP.

## Data Availability

The datasets generated for this study are available on request from the corresponding author.

## Ethics Statement

All procedures and experiments involving mice were carried out with approval of the University of Iowa Institutional Animal Care and Use Committee, and in strict accordance with the recommendations of the ACP Guide for the Care and Use of Laboratory Animals (2011).

## Author Contributions

FT, YK, MC, KE, and GR were responsible for the collection and analysis of data. FT, YK, and GR were responsible for the conception and design of the experiments. FT drafted the manuscript. All authors were responsible for interpretation of the data and revised the manuscript critically for important intellectual content. All authors have approved the final version of the manuscript and agree to be accountable for all aspects of the work. All persons designated as authors qualify for authorship, and all those who qualify for authorship are listed.

### Conflict of Interest Statement

The authors declare that the research was conducted in the absence of any commercial or financial relationships that could be construed as a potential conflict of interest.
